# Ces3/TGH Deficiency Attenuates Steatohepatitis

**DOI:** 10.1038/srep25747

**Published:** 2016-05-16

**Authors:** Jihong Lian, Enhui Wei, Jody Groenendyk, Subhash K. Das, Martin Hermansson, Lena Li, Russell Watts, Aducio Thiesen, Gavin Y. Oudit, Marek Michalak, Richard Lehner

**Affiliations:** 1 Group on Molecular and Cell Biology of Lipids, University of Alberta, Edmonton, Alberta, Canada; 2 Department of Pediatrics, Faculty of Medicine and Dentistry, University of Alberta, Edmonton, Alberta, Canada; 3 Department of Biochemistry, Faculty of Medicine and Dentistry, University of Alberta, Edmonton, Alberta, Canada; 4 Department of Medicine, Faculty of Medicine and Dentistry, University of Alberta, Edmonton, Alberta, Canada; 5 Department of Pathology, Faculty of Medicine and Dentistry, University of Alberta, Edmonton, Alberta, Canada; 6 Department of Cell Biology, Faculty of Medicine and Dentistry, University of Alberta, Edmonton, Alberta, Canada

## Abstract

Nonalcoholic fatty liver disease (NAFLD) is the most common form of chronic liver disease in developed countries. NAFLD describes a wide range of liver pathologies from simple steatosis to nonalcoholic steatohepatitis (NASH) and cirrhosis. NASH is distinguished from simple steatosis by inflammation, cell death and fibrosis. In this study we found that mice lacking triacylglycerol hydrolase (TGH, also known as carboxylesterase 3 or carboxylesterase 1d) are protected from high-fat diet (HFD) - induced hepatic steatosis via decreased lipogenesis, increased fatty acid oxidation and improved hepatic insulin sensitivity. To examine the effect of the loss of TGH function on the more severe NAFLD form NASH, we ablated *Tgh* expression in two independent NASH mouse models, *Pemt*^*−/−*^ mice fed HFD and *Ldlr*^*−/−*^ mice fed high-fat, high-cholesterol Western-type diet (WTD). TGH deficiency reduced liver inflammation, oxidative stress and fibrosis in *Pemt*^*−/−*^ mice. TGH deficiency also decreased NASH in *Ldlr*^*−/−*^ mice. Collectively, these findings indicate that TGH deficiency attenuated both simple hepatic steatosis and irreversible NASH.

Nonalcoholic fatty liver disease (NAFLD) is recognized as the leading cause of chronic liver injury in Western societies. It is commonly associated with insulin resistance, type 2 diabetes and cardiovascular disease. Clinical phonotypes of NAFLD extend from simple steatosis, which is characterized by excess deposition of triacylglycerol (TG) in the liver, to nonalcoholic steatohepatitis (NASH), which is distinguished from steatosis by the presence of hepatocyte injury (ballooning and cell death), inflammation and/or fibrosis. NASH can further progress to liver cirrhosis and hepatocellular carcinoma^1^,^2^.

Mouse carboxylesterases have been shown to participate in hepatic lipid metabolism, including carboxylesterase 3 (Ces3)^3^,^4^,^5^ and carboxylesterase 1 (Ces1)^6^,^7^. Mouse Ces3 is also annotated as triacylglycerol hydrolase (TGH) or Ces1d, while Ces1 is also annotated as esterase-x (Es-x) or Ces1 g^8^. The human ortholog of mouse Ces3/TGH/Ces1d is CES1^8^. The human ortholog of mouse Ces1/Es-x/Ces1 g has not yet been defined. Some reports used the human nomenclature CES1 also for mouse Ces1/Es-x/Ces1 g^9^,^7^,^10^. However, it is important to distinguish between Ces3/TGH/Ces1d and Ces1/Es-x/Ces1 g because these two carboxylesterases play very different metabolic functions^6^,^11^. We will refer to CES1/Ces3/Ces1d/TGH as TGH in this study. TGH participates in basal lipolysis in adipocytes^12^,^13^. In the liver, TGH is involved in the provision of substrates for the assembly of hepatic very low-density lipoproteins (VLDL) and inhibition of TGH decreased VLDL secretion both *in vitro* and *in vivo*^4^,^14^,^15^. Blocking hepatic VLDL assembly and secretion can lead to severe hepatic lipid accumulation^16^, but this condition was prevented in TGH deficient (*Tgh*^*−/−*^) mice due to the compensatory effect of decreased non-esterified fatty acid (NEFA) flux from the adipose tissue to liver and increased hepatic fatty acid oxidation^15^,^17^. In humans, elevated *TGH* expression was observed in patients with steatosis and NASH^18^. However, the role of TGH in NAFLD development has not been determined.

Phosphatidylethanolamine *N*-methyltransferase (PEMT) catalyzes the conversion of phosphatidylethanolamine (PE) to phosphatidylcholine (PC), which is quantitatively important in the liver PC synthesis^19^,^20^. Loss-of-function single nucleotide polymorphism in human PEMT is correlated with increased risk of NAFLD^21^. *Pemt*^*−/−*^ mice are susceptible to diet-induced liver steatosis^22^ and NASH. One week of high-fat diet (HFD) feeding of *Pemt*^*−/−*^ mice was sufficient to induce liver steatosis and NASH features including inflammation and oxidative stress^22^, and prolonged HFD feeding period (10 weeks) resulted in severe liver damage in *Pemt*^*−/−*^ mice^23^. The development of steatosis in *Pemt*^*−/−*^ mice is likely to due to the reduction of PC required for VLDL assembly. Aberrant PC homeostasis and decreased PC to PE ratio caused by PEMT deficiency leads to impairment of plasma membrane integrity and leakage of hepatocellular content into the extracellular space, which can provoke NASH features such as inflammation and hepatocyte injury^20^,^22^,^24^. NASH also develops in LDL receptor knockout (*Ldlr*^*−/−*^) mice fed high-fat, high-cholesterol Western-type diet (WTD)^25^,^26^. The current study suggests that the loss of TGH function is protective against development of HFD-induced hepatic steatosis and also ameliorates NASH in *Pemt*^*−/−*^ and *Ldlr*^*−/−*^ mice.

## Results

### Absence of TGH Attenuates HFD Induced Hepatic Steatosis

**Figure 1:**
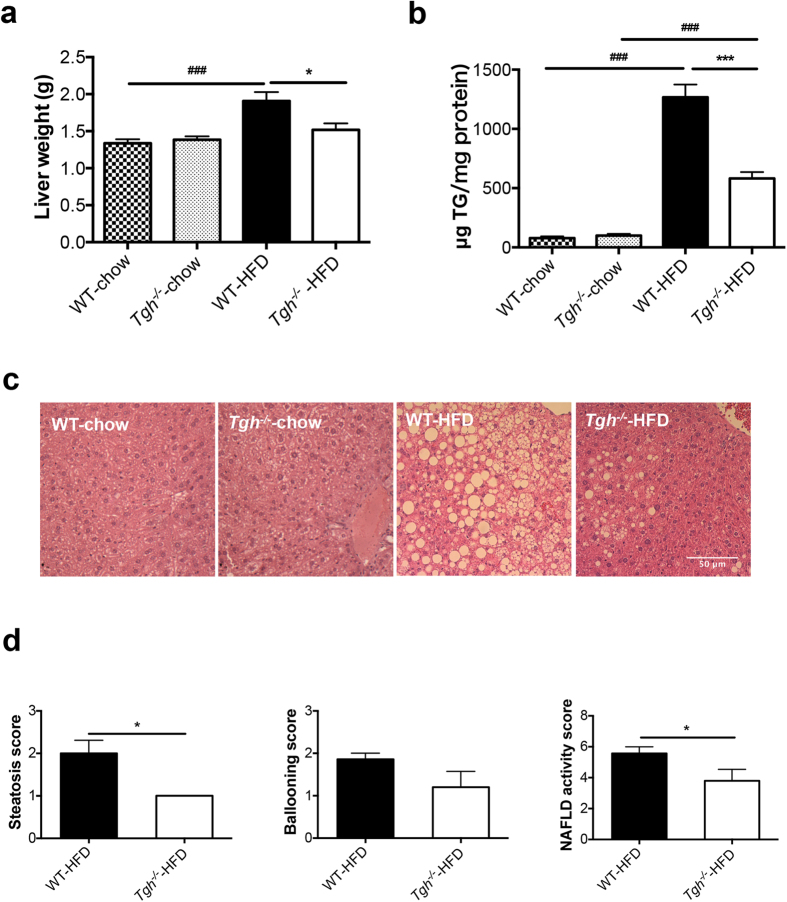
TGH deficiency attenuates diet induced liver steatosis. (**a**) Liver weight of WT and *Tgh*^*−/−*^ mice on chow and HFD (n = 5–7). (**b**) Hepatic TG mass (n = 5–7). (**c**) Liver slices were stained with hematoxylin and eosin (n = 5). (**d**) Liver histology was clinically assessed for steatosis, ballooning, and NAFLD activity scores. Data are mean ± SEM, *P < 0.05, **P < 0.01, ***P < 0.001 vs WT mice on the same diet condition, ^###^*P* < 0.001 vs chow diet fed mice in the same genotype.

*Tgh*^*−/−*^ mice on chow diet showed decreased plasma TG with unaltered liver weight and absence of liver TG accumulation (Supplementary Table 1, Fig. 1a,b). No histological differences in the liver were observed between chow fed *Tgh*^*−/−*^ and WT mice (Fig. 1c).

**Figure 2:**
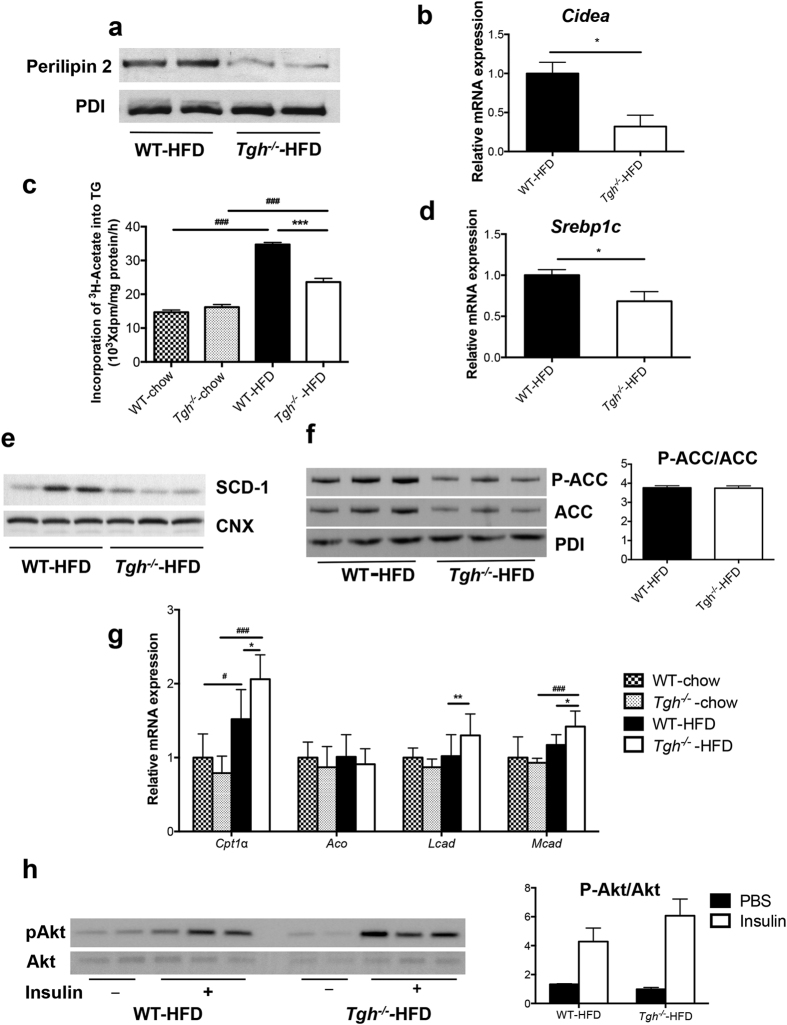
TGH deficiency ameliorates steatosis through various metabolic pathways. (**a**) Perilipin 2 abundance evaluated by immunoblot in HFD fed WT and *Tgh*^*−/−*^ mice, Immunoblotting for PDI was used as control for protein loading. (**b**) mRNA expression of *Cidea* in HFD fed WT and *Tgh*^*−/−*^ mice (n = 5). (**c**) *De novo* lipogenesis was assessed by synthesis of FA from [^3^H]acetic acid that are incorporated into TG in primary hepatocytes isolated from chow and HFD fed WT and *Tgh*^*−/−*^ mice. (**d**) mRNA expression of *Srebf1c*. (**e**) Liver SCD-1 abundance in HFD fed WT and *Tgh*^*−/−*^ mice was assessed by immunoblotting. Calnexin immunoblotting was used as control for protein loading. (**f**) Immunoblotting for liver phospho-(Ser79) acetyl-CoA carboxylase (ACC) and total ACC in HFD fed WT and *Tgh*^*−/−*^ mice. Immunoreactive bands were quantified by the densitometric analysis and the p-ACC/ACC ratio was calculated. (**g**) mRNA expression of genes related to FA oxidation (n = 5,6). (**h**) Hepatic *In vivo* insulin signaling. Immunoreactive bands were quantified by the densitometric analysis and the pAkt/total-Akt ratio was calculated in each condition. Data are mean ± SEM, *P < 0.05, **P < 0.01, ***P < 0.001 vs WT group on the same diet condition, ^#^*P* < 0.05, ^##^*P* < 0.01, ^###^*P* < 0.001 vs chow diet fed group in the same genotype.

Both *Tgh*^*−/−*^ and control wild-type (WT) mice exhibited similar increase in weight gain when fed HFD for 16 weeks (Supplementary Table 1). As expected, HFD feeding in WT mice increased liver weight and liver TG accumulation (Fig. 1a,b). However, reduction of liver TG mass by 54.1% was observed in *Tgh*^*−/−*^ mice fed HFD when compared with WT mice on the same diet. Importantly, while liver weight of WT mice fed HFD significantly increased, liver weight of *Tgh*^*−/−*^ mice fed HFD did not statistically differ from liver weights of WT or *Tgh*^*−/−*^ mice fed chow diet (Fig. 1a). Consequently, liver histology showed steatosis in HFD fed WT mice, whereas TGH deficiency ameliorated the pathology (Fig. 1c,d). Hepatic free fatty acid (FFA) concentration in HFD fed *Tgh*^*−/−*^ mice showed a decreased trend (*p* = 0.077) (Supplementary Fig. 1). Perilipin 2, a lipid droplet (LD)-associated protein, was reduced in the liver of *Tgh*^*−/−*^ mice fed a HFD (Fig. 2a). Increased mRNA expression of *CIDEA* is positively correlated with the severity of hepatic steatosis in humans^27^. Expression of *Cidea* was significantly reduced in the livers of *Tgh*^*−/−*^ mice fed HFD (Fig. 2b).

Hepatic *de novo* lipogenesis is an important contributing factor to hepatic steatosis and the role of FA synthesis is especially critical in HFD fed condition^28^,^29^. In agreement with the reported effect of HFD feeding on lipogenesis^28^, *de novo* TG synthesis assessed by acetate incorporation was increased by 136% in hepatocytes from WT mice fed a HFD when compared to chow fed WT mice and this increase was significantly reduced by the absence of TGH (Fig. 2c). This reduction of lipogenesis in *Tgh*^*−/−*^ mice after HFD could be in part attributed to reduced expression of *Srebf1c* (Fig. 2d) and its key target stearoyl-CoA desaturase-1 (SCD1) (Fig. 2e). Similarly, the abundance of another *Srebf1c* target acetyl-CoA carboxylase (ACC) was reduced in *Tgh*^*−/−*^ livers, while the ratio of phospho-(Ser79) ACC to total ACC was not affected (Fig. 2f). Hepatic expression of FA oxidation related genes *Cpt1*α, *Lcad*, and *Mcad* were increased in HFD fed *Tgh*^*−/−*^ mice (Fig. 2g), suggesting increased FA oxidation.

Because augmented lipogenesis after HFD feeding is often associated with impaired hepatic insulin sensitivity^30^,^31^, *in vivo* insulin signaling assay was performed in mice after HFD feeding. Compared to the WT group, livers of *Tgh*^*−/−*^ mice showed increased insulin-stimulated Akt phosphorylation, suggesting improved insulin sensitivity (Fig. 2h).

### Decreased VLDL-TG Production in *Tgh^−/−^/Pemt^−/−^* Mice

**Figure 3:**
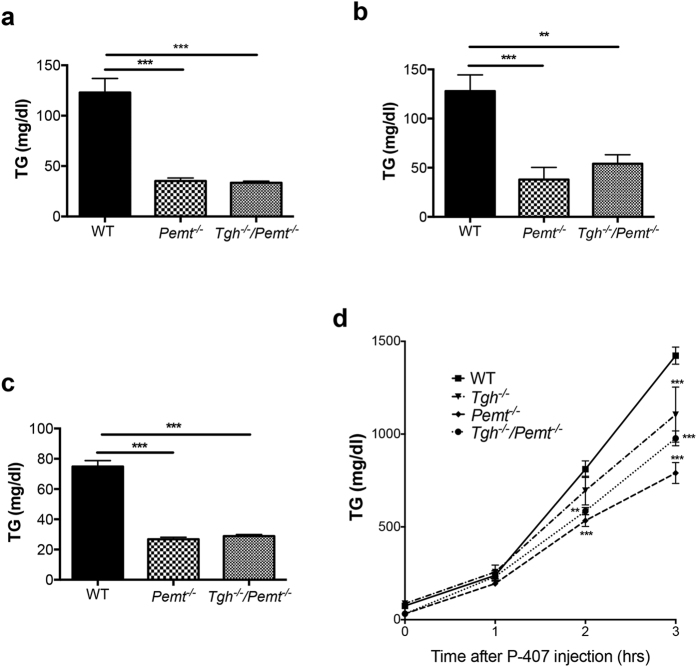
VLDL production and plasma TG concentrations in *Pemt*^*−/−*^ and *Tgh*^*−/−*^*/Pemt*^*−/−*^ mice fed chow and HFD. (**a**) Plasma TG levels in WT, *Pemt*^*−/−*^, and *Tgh*^*−/−*^*/Pemt*^*−/−*^ mice fed chow. (**b**) Plasma TG levels in WT, *Pemt*^*−/−*^, and *Tgh*^*−/−*^*/Pemt*^*−/−*^ mice after 1 week of HFD. (**c**) Plasma TG levels in WT, *Pemt*^*−/−*^, and *Tgh*^*−/−*^*/Pemt*^*−/−*^ mice after 10 weeks of HFD. (**d**) *In vivo* VLDL-TG secretion in WT, *Tgh*^*−/−*^, *Pemt*^*−/−*^, and *Tgh*^*−/−*^*/Pemt*^*−/−*^ mice after 10 weeks of HFD. n = 5,6. Data are mean ± SEM, **P < 0.01, ***P < 0.001 vs WT control.

To investigate whether TGH deficiency can prevent NASH, a more severe form of hepatic steatosis, we crossed *Tgh*^*−/−*^ mice with *Pemt*^*−/−*^ mice (Supplementary Fig. 2). *Pemt*^*−/−*^ mice exhibited dramatically decreased plasma TG when fed chow diet (Fig. 3a), after short-term (1 week) HFD feeding (Fig. 3b), and after long-term (10 weeks) HFD feeding (Fig. 3c). *Tgh*^*−/−*^*/Pemt*^*−/−*^ mice fed chow and HFD showed similar decrease of plasma TG concentrations as *Pemt*^*−/−*^ mice on the same diet condition (Fig. 3a–c). Reduced VLDL-TG secretion rate *in vivo* was observed in 10-week HFD fed *Tgh*^*−/−*^, *Pemt*^*−/−*^ and *Tgh*^*−/−*^*/Pemt*^*−/−*^ mice (Fig. 3d).

### TGH Deficiency Attenuates NASH in *Pemt^−/−^* Mice

**Figure 4:**
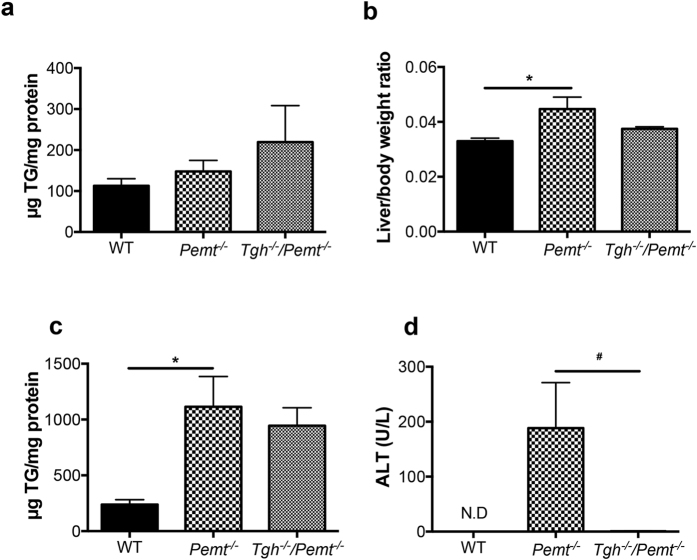
Hepatic metabolic parameters in *Pemt*^*−/−*^ and *Tgh*^*−/−*^*/Pemt*^*−/−*^ mice fed chow and 1 week HFD. (**a**) Liver TG mass in WT, *Pemt*^*−/−*^ and *Tgh*^*−/−*^*/Pemt*^*−/−*^ mice fed chow. (**b**) Liver/ body weight ratio in WT, *Pemt*^*−/−*^ and *Tgh*^*−/−*^*/Pemt*^*−/−*^ mice after 1 week HFD. (**c**) Liver TG mass in WT, *Pemt*^*−/−*^ and *Tgh*^*−/−*^*/Pemt*^*−/−*^ mice after 1 week HFD. (**d**) Plasma ALT in WT, *Pemt*^*−/−*^ and *Tgh*^*−/−*^*/Pemt*^*−/−*^ mice after 1 week HFD. n = 5,6. Data are mean ± SEM. *P < 0.05 vs WT control, ^#^P < 0.05 vs *Pemt*^*−/−*^ mice.

While *Pemt*^*−/−*^ and *Tgh*^*−/−*^*/Pemt*^*−/−*^ mice contained comparable TG concentration in livers as WT mice when fed a chow diet (Fig. 4a), one week of HFD feeding significantly increased liver weights and TG mass in *Pemt*^*−/−*^ mice (Fig. 4b,c). Although liver weight was reduced in *Tgh*^*−/−*^*/Pemt*^*−/−*^ mice, TG mass did not change significantly compared with *Pemt*^*−/−*^ mice after 1 week HFD (Fig. 4b,c). Liver cholesterol levels also did not differ between *Pemt*^*−/−*^ and *Tgh*^*−/−*^*/Pemt*^*−/−*^ mice (Supplementary Fig. 3), although the expression of key genes regulating sterol synthesis was decreased by TGH deficiency (Supplementary Fig. 4). However, the dramatically elevated plasma alanine-amino transferase (ALT) in *Pemt*^*−/−*^ mice was not observed in *Tgh*^*−/−*^*/Pemt*^*−/−*^ mice indicating absence of liver damage in *Tgh*^*−/−*^*/Pemt*^*−/−*^ mice (Fig. 4d).

**Figure 5:**
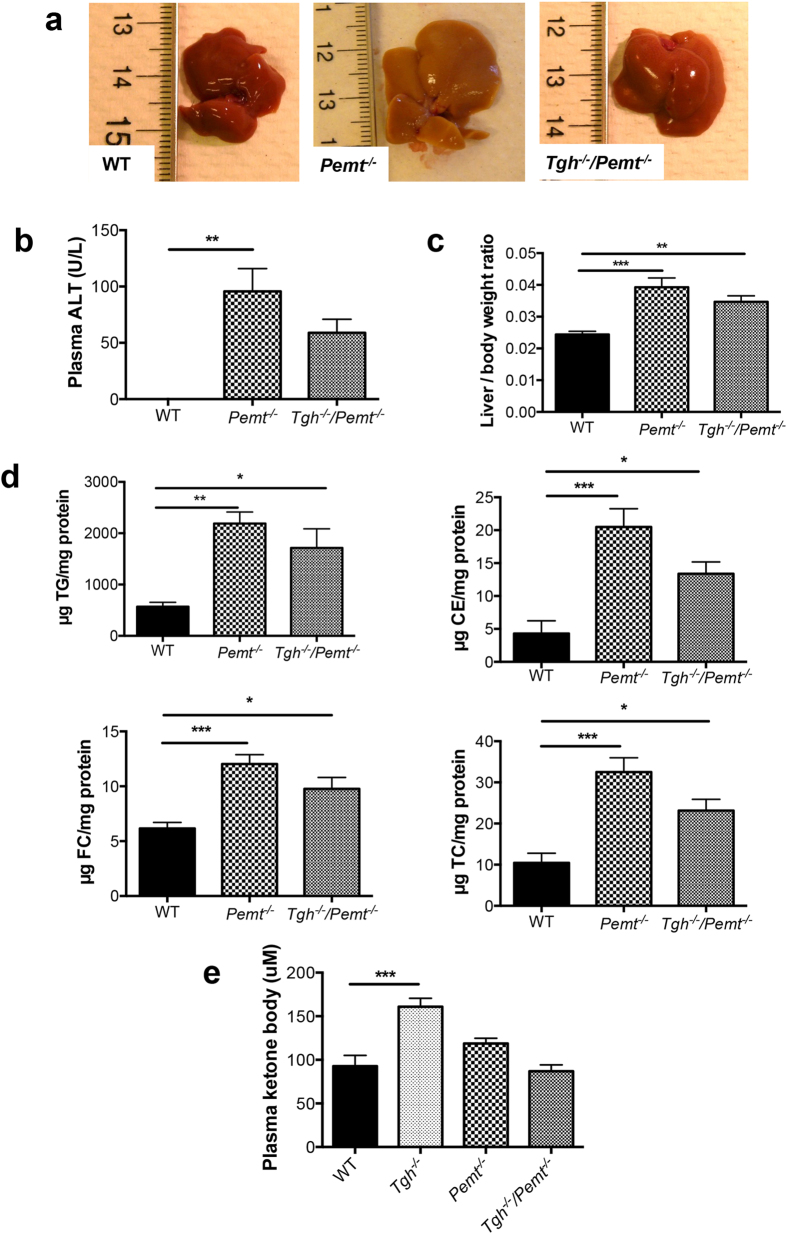
Hepatic metabolic parameters in *Pemt*^*−/−*^ and *Tgh*^*−/−*^*/Pemt*^*−/−*^ mice fed HFD for 10 weeks. (**a**) Livers from WT, *Pemt*^*−/−*^ and *Tgh*^*−/−*^*/Pemt*^*−/−*^ mice. (**b**) Plasma ALT in WT, *Pemt*^*−/−*^ and *Tgh*^*−/−*^*/Pemt*^*−/−*^ mice. (**c**) Liver/ body weight ratio in WT, *Pemt*^*−/−*^, and *Tgh*^*−/−*^*/Pemt*^*−/−*^ mice. (**d**) Liver TG, CE, FC, and TC mass in WT, *Pemt*^*−/−*^, and *Tgh*^*−/−*^*/Pemt*^*−/−*^ mice. (**e**) Plasma ketone bodies concentrations in WT, *Tgh*^*−/−*^, *Pemt*^*−/−*^ and *Tgh*^*−/−*^*/Pemt*^*−/−*^ mice. n = 5, 6. Data are mean ± SEM. *P < 0.05, **P < 0.01, ***P < 0.001 vs WT control.

To investigate the effect of TGH deficiency on NASH development, we prolonged the HFD feeding regimen to 10 weeks to induce more advanced steatohepatitis in *Pemt*^*−/−*^ mice^23^. As expected, *Pemt*^*−/−*^ mice exhibited liver damage after 10 weeks HFD feeding, whereas the less pale liver appearance and decreased trend of plasma ALT in *Tgh*^*−/−*^*/Pemt*^*−/−*^mice suggested amelioration of the condition (Fig. 5a,b). After 10 weeks HFD feeding PEMT deficiency increased the liver to body weight ratio by 61%, elevated liver TG, cholesteryl ester (CE), free cholesterol (FC), and total cholesterol (TC) by 286%, 376%, 95%, and 211% respectively, compared with the WT group. *Tgh*^*−/−*^*/Pemt*^*−/−*^mice did not show significantly reduced liver lipids when compared with *Pemt*^*−/−*^ mice (Fig. 5c,d). No difference in liver FFA was observed among all groups (Supplementary Fig. 5). Interestingly, increased concentration of ketone bodies observed in plasma of HFD fed *Tgh*^*−/−*^ mice was attenuated in *Tgh*^*−/−*^*/Pemt*^*−/−*^mice (Fig. 5e). No changes in body weight were observed between *Pemt*^*−/−*^ and *Tgh*^*−/−*^*/Pemt*^*−/−*^ mice (Supplementary Fig. 6).

**Figure 6:**
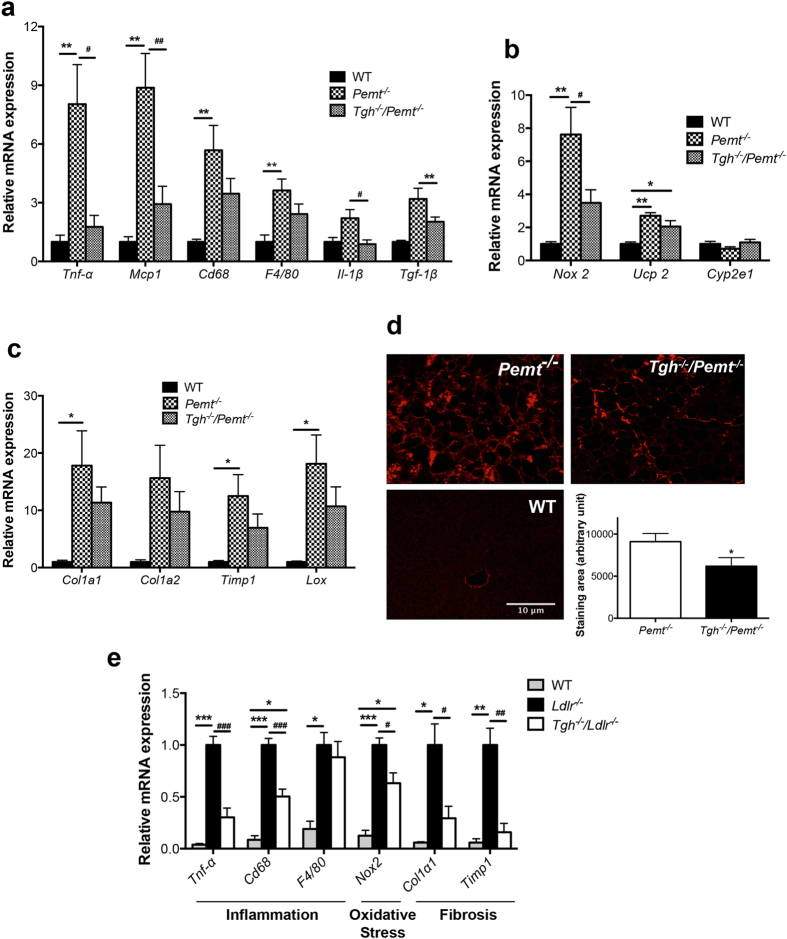
TGH deficiency ameliorates NASH features in *Pemt*^*−/−*^ mice and *Ldlr*^*−/−*^ mice. (**a**) qPCR analysis of inflammatory genes expression in livers of WT, *Pemt*^*−/−*^ and *Tgh*^*−/−*^*/Pemt*^*−/−*^ mice after 10 weeks of HFD feeding, (**b**) qPCR analysis of oxidative stress genes expression in livers of WT, *Pemt*^*−/−*^ and *Tgh*^*−/−*^*/Pemt*^*−/−*^ mice, (**c**) qPCR analysis of fibrosis genes expression in livers of WT, *Pemt*^*−/−*^ and *Tgh*^*−/−*^*/Pemt*^*−/−*^ mice. n = 5. Data are mean ± SEM. *P < 0.05, **P < 0.01 vs WT control, ^*#*^P < 0.05, ^*##*^P < 0.01  vs *Pemt*^*−/−*^ mice. (**d**) Representative picrosirius red (PSR)-stained liver sections, and averaged collagen volume fraction in *Pemt*^*−/−*^ and *Tgh*^*−/−*^*/Pemt*^*−/−*^ mice. n = 5. Data are mean ± SEM. *P < 0.05, vs *Pemt*^*−/−*^ mice. (**e**) qPCR analysis of representative NASH genes in livers of *Ldlr*^*−/−*^ and *Tgh*^*−/−*^*/Ldlr*^*−/−*^ after 12 weeks of WTD. n = 5. Data are mean ± SEM. *P < 0.05, **P < 0.01, ***P < 0.001 vs *Ldlr*^*−/−*^ mice.

Liver inflammation is an important feature of NASH. After 10 weeks on a HFD, *Pemt*^*−/−*^ mice exhibited elevated hepatic expression of proinflammatory cytokines, including tumor necrosis factor alpha (TNF-α), monocyte chemotactic protein-1 (MCP-1), interleukin (IL)-6, IL-1β, and transforming growth factor (TGF)-1β. Increased expression of macrophage markers cluster of differentiation 68 (CD68) and F4/80 were also observed in *Pemt*^*−/−*^ livers (Fig. 6a). TGH deficiency significantly attenuated hepatic expression of these inflammatory markers and cytokines in *Pemt*^*−/−*^ mice (Fig. 6a). Oxidative stress has been implicated in the progression of NASH^32^. Ten-week HFD feeding of *Pemt*^*−/−*^ mice resulted in increased expression of oxidative stress markers NADPH oxidase 2 (NOX 2) and uncoupling protein-2 (UCP-2) in the liver and the expression of these markers was significantly decreased by TGH deficiency (Fig. 6b).

Because inflammatory cytokines including MCP-1, IL-1β and TGF-1β are related to hepatic stellate cell activation and fibrogenesis^33^,^34^, expression of liver fibrosis markers was also determined. High hepatic expression of fibrillar extracellular matrix (ECM) collagen type 1 (COL1A1 and COL1A2) was observed in *Pemt*^*−/−*^ mice. Tissue inhibitor of metalloproteinase1 (TIMP1), which is capable of inactivating collagenase, and lysyl oxidase (LOX), which is a key enzyme promoting ECM intermolecular cross-linking and was implicated in liver fibrosis^35^, were also significantly higher in livers of *Pemt*^*−/−*^ mice compared to control mice (Fig. 6c). In *Tgh*^*−/−*^*/Pemt*^*−/−*^mice, expression of all tested fibrosis markers showed decreased trends (Fig. 6c). Consequently, severe liver fibrosis observed in *Pemt*^*−/−*^ mice was diminished by TGH deficiency (Fig. 6d).

### TGH Deficiency Attenuates NASH Features in *Ldlr^−/−^* Mice

To investigate if the attenuation of NASH features by TGH deficiency was animal model independent, we used another NASH mouse model. Unlike the hypolipidemic *Pemt*^*−/−*^ mice, *Ldlr*^*−/−*^ mice develop severe hyperlipidemia and NASH after 12 weeks of WTD feeding^36^. Liver TG and CE mass were not different between *Ldlr*^*−/−*^ and *Tgh*^*−/−*^*/Ldlr*^*−/−*^ mice^37^, nor was liver FFA concentration (Supplementary Fig. 7). However, when compared to *Ldlr*^*−/−*^ mice, *Tgh*^*−/−*^*/Ldlr*^*−/−*^ mice after WTD feeding exhibited lower expression of inflammatory, oxidative stress and fibrosis markers in the liver, implying ameliorated liver pathophysiology (Fig. 6e).

### TGH Deficiency Partially Restores Aberrant Phospholipid Composition in *Pemt^−/−^* Mice

**Figure 7:**
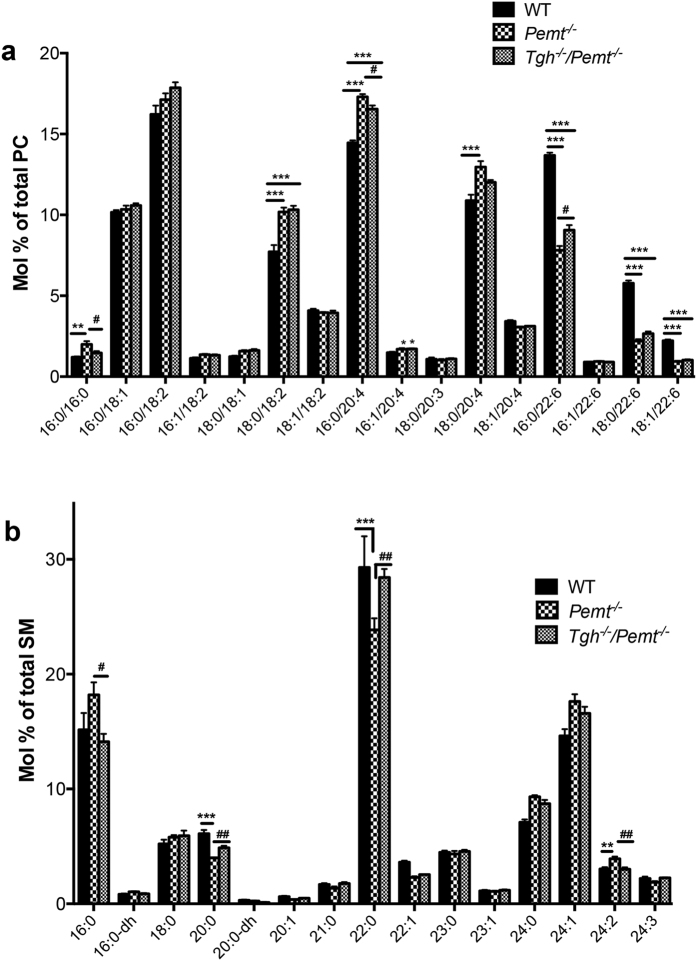
Partial restoration of phospholipid molecular species by TGH deficiency in *Pemt*^*−/−*^ mice fed HFD for 10 weeks. (**a**) Liver PC molecular species in WT, *Pemt*^*−/−*^ and *Tgh*^*−/−*^*/Pemt*^*−/−*^ mice. (**b**) Liver SM molecular species in WT, *Pemt*^*−/−*^ and *Tgh*^*−/−*^*/Pemt*^*−/−*^ mice. n = 5. Data are mean ± SEM. **P < 0.01, ***P < 0.001 vs WT control, ^#^P < 0.05, ^##^P < 0.01 vs *Pemt*^*−/−*^ mice.

Decreased hepatic PC to PE ratio in PEMT deficient mice leads to impaired lipid bilayer integrity and consequently liver damage and inflammation^20^,^22^,^24^. Ten weeks of HFD feeding decreased the PC to PE ratio by 38% in *Pemt*^*−/−*^ mice (Supplementary Fig. 8a). TGH deficiency in *Pemt*^*−/−*^ mice increased liver PC mass (Supplementary Fig. 8b), however, no significant difference in hepatic PC to PE ratio was observed between *Pemt*^*−/−*^ and *Tgh*^*−/−*^*/Pemt*^*−/−*^ mice (Supplementary Fig. 8a), suggesting that ablation of *Tgh* expression provides protection against inflammation and liver damage by alternative mechanisms than by increasing PC abundance. The PEMT pathway contributes polyunsaturated fatty acid (PUFA) species to liver PC, mainly docosahexaenoic acid (DHA, 22:6n−3)^19^. Changes in hepatic PC-PUFA composition were observed in *Pemt*^*−/−*^ mice after 10 weeks of HFD feeding, yielding elevated arachidonic acid (ARA, 20:4n-6) containing PC and decreased DHA containing PC. TGH deficiency in *Pemt*^*−/−*^ mice decreased ARA and increased DHA in PC (Fig. 7a, Supplementary Fig. 9). In addition, increased dipalmitoyl-PC (C16:0/C16:0) concentration in the *Pemt*^*−/−*^ livers was reduced by TGH deficiency (Fig. 7a). Increased sphingomyelin (SM) abundance, particularly of species containing saturated fatty acids, is associated with liver steatosis. Increased concentration of palmitoylsphingomyelin (16:0-SM) was observed in the livers of 10-week HFD fed *Pemt*^*−/−*^ mice and was normalized to the level of the WT group in the *Tgh*^*−/−*^*/Pemt*^*−/−*^ mice (Fig. 7b). Additionally, reduction of 20:0 and 22:0-SM in *Pemt*^*−/−*^ mouse liver was reversed by TGH deficiency. No differences were observed in hepatic TG FA composition between *Tgh*^*−/−*^ and *Tgh*^*−/−*^*/Pemt*^*−/−*^ mice (Supplementary Fig. 10).

### TGH deficiency decreases circulating cytokines in *Pemt^−/−^* mice

**Figure 8:**
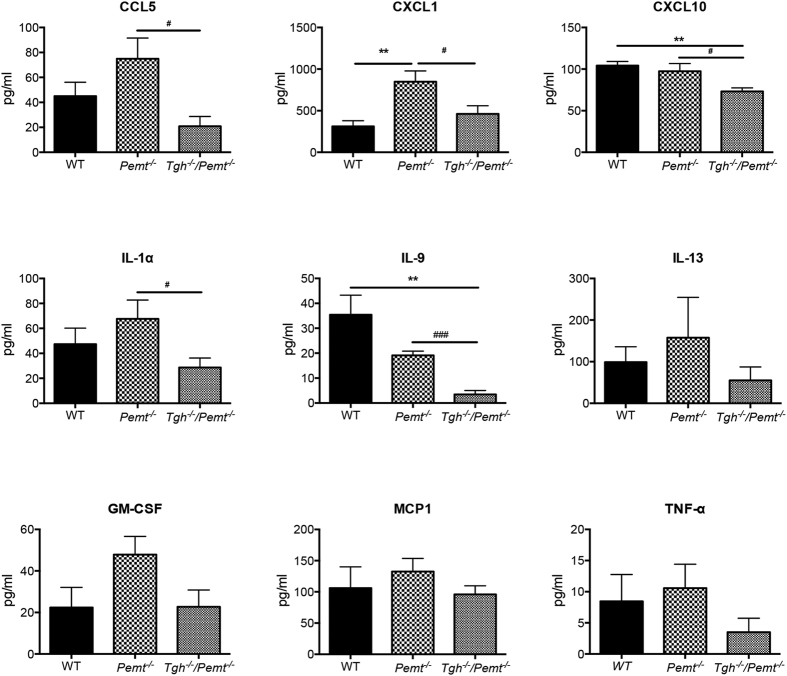
TGH deficiency decreases plasma cytokines levels in *Pemt*^*−/−*^ mice. n = 5. Data are mean mean ± SEM. *P < 0.05, **P < 0.01 vs WT mice, ^#^P < 0.05, ^###^ < 0.001 vs *Pemt*^*−/−*^ mice.

A variety of cytokines (CCL5, CXCL1, CXCL10, IL-1α, IL-9, IL-13, GM-CSF, MCP-1, and TNF-α) that promote liver inflammation and fibrosis, or are upregulated in NASH patients^38^,^39^,^40^,^41^, were measured in plasma of 10-week HFD fed WT, *Pemt*^*−/−*^ and *Tgh*^*−/−*^*/Pemt*^*−/−*^ mice. CXCL1 was significantly increased in *Pemt*^*−/−*^ mice when compared with WT and ablating *Tgh* expression in *Pemt*^*−/−*^ mice resulted in decreased concentration in this cytokine to levels comparable with WT mice (Fig. 8). Additionally, CCL5, CXCL10, IL-1α, and IL-9 concentrations were diminished in *Tgh*^*−/−*^*/Pemt*^*−/−*^ mice compared with *Pemt*^*−/−*^ mice to levels comparable with or lower than (CXCL10, IL-9) WT mice (Fig. 8). Therefore, *d*ecreased circulating cytokines may have also contributed to the attenuated NASH development in *Tgh*^*−/−*^*/Pemt*^*−/−*^ mice.

### NASH in *Pemt^−/−^* Mice Was Not Accompanied by Aggravated Hepatic Insulin Resistance

Compared with the WT group, both *Pemt*^*−/−*^ and *Tgh*^*−/−*^*/Pemt*^*−/−*^ groups exhibited increased trend of Akt activation by phosphorylation (Supplementary Fig. 11). This result implied dissociation of hepatic insulin resistance and NASH in *Pemt*^*−/−*^ mice, which is consistent with previous findings^23^.

### TGH deficiency did not affect ER stress in *Pemt^−/−^* mice

ER dysfunction is related to hepatic steatosis progression and the development of NASH features such as inflammation, hepatocyte injury and oxidative stress^42^,^43^. ER stress markers and sensors have been assessed including calreticulin and BiP^44^, activating transcription factor (ATF) 6 and inositol-requiring enzyme (IRE) 1α^45^, total and spliced X-box binding protein-1 (XBP1)^46^ (Supplementary Fig. 12). Only total and spliced XBP1 mRNA showed increased abundance in HFD fed groups when compared with chow fed WT control but no differences in HFD-induced Xbp1 and spliced XBP1 mRNA were observed among the HFD-fed groups.

## Discussion

The clinical and histological spectrum of NAFLD ranges from reversible simple steatosis to NASH, which is characterized by liver damage, inflammatory infiltrate and/or collagen deposition (fibrosis). NASH can progress to cirrhosis and hepatocellular carcinoma^1^,^2^.

NAFLD is commonly associated with severe obesity and various metabolic disorders such as insulin resistance, type 2 diabetes, dyslipidemia and cardiovascular disease. However, NAFLD can also occur in normolipidemic patients that are not obese or diabetic^47^. In the current study, we have interrogated the role of TGH in NAFLD progression. Chow fed TGH deficient mice are protected from hepatic lipid accumulation despite decreased VLDL secretion^15^. Therefore, we investigated whether TGH deficiency would be protective against HFD-induced hepatic steatosis in a diet-induced obese mouse model and found that after 16 weeks HFD feeding *Tgh*^*−/−*^ mice had decreased liver lipid mass and steatosis severity when compared with WT mice. Patients afflicted with NAFLD present with elevated *de novo* lipogenesis, which contributes to hepatic lipid accumulation^48^. Decreased *de novo* lipogenesis was observed in *Tgh*^*−/−*^ mice, as a result of decreased SREBP1c pathway. Augmented FA oxidation and improved insulin sensitivity may have additionally contributed to protection from steatosis.

To investigate whether TGH deficiency would attenuate progression to the inflammatory steatohepatitis, we used two different NASH mouse models: *Pemt*^*−/−*^ mice fed HFD for 10 weeks and *Ldlr*^*−/−*^ mice fed WTD for 12 weeks. TGH deficiency ameliorated NASH features including inflammation, oxidative stress and fibrosis in both of these animal models.

*Pemt*^*−/−*^ mouse fed HFD for 10 weeks is a NASH model that is disassociated from insulin resistance and hyperlipidemia^23^. Unlike the significantly decreased steatosis in HFD fed *Tgh*^*−/−*^ mice, *Tgh*^*−/−*^*/Pemt*^*−/−*^ mice contained similar liver lipid levels as *Pemt*^*−/−*^ mice. Hepatic protein expression of two key enzymes in the *de novo* lipogenesis, SCD-1 and fatty acid synthase (FAS), was decreased in *Pemt*^*−/−*^ mice when compared with WT mice^23^ and additional reduction was not observed in *Tgh*^*−/−*^/*Pemt*^*−/−*^ mice. Furthermore, hepatic insulin signaling remains normal in HFD fed *Pemt*^*−/−*^ mice despite profound steatosis with NASH features and TGH deficiency did not further affect insulin signaling. Although TGH deficiency alone increased plasma ketone bodies concentration, *Tgh*^*−/−*^*/Pemt*^*−/−*^ mice had similar plasma ketone bodies levels as *Pemt*^*−/−*^ and WT mice.

One potential mechanism by which TGH deficiency attenuates NASH progression in *Pemt*^*−/−*^ mice is through partial reconstitution of phospholipid molecular species in the liver. Decreased DHA-PC and increased dipalmitoyl-PC may have contributed to the decreased fluidity and dysfunction of cell membranes observed in NASH induced in *Pemt*^*−/−*^ mice^24^. By normalizing the FA composition of PC, TGH deficiency restored the membrane disorder found in *Pemt*^*−/−*^ mice and attenuated inflammation and liver damage leading to NASH.

In the absence of the PEMT pathway PE is converted to TG through phospholipase C and D pathways^49^ yielding increased PUFA content in TG. In this study increased hepatic PUFA in TG was also observed in *Tgh*^*−/−*^ mice, which might have been due to decreased TG turnover of these species. However, further increment of PUFA in liver TG was not observed in *Tgh*^*−/−*^*/Pemt*^*−/−*^ mice, suggestive of an alternative pathway that channels PUFA-rich metabolites derived from PE. In our previous study^50^, increased PC synthesis through CDP-choline pathway was observed in *Tgh*^*−/−*^ hepatocytes, which may have normalized FA composition in liver PC in *Tgh*^*−/−*^*/Pemt*^*−/−*^ mice.

Importantly, it has been shown that hepatic TGH expression is regulated by IL-6, TGF-β, and TNF-α^51^, thus implying the general relationship between TGH activity and inflammation. However, detailed mechanism of how TGH regulates inflammation still remains to be elucidated. Notably, the present study showed that although circulating cytokines were not comprehensively increased in *Pemt*^*−/−*^ mice when compared with WT mice, TGH deficiency reduced circulating concentrations of several cytokines in *Pemt*^*−/−*^ mice after 10 weeks HFD, which may have also contributed to the amelioration of NASH in the *Tgh*^*−/−*^*/Pemt*^*−/−*^ mice. Because TGH is expressed in both adipose tissue and liver, tissue specific contribution of TGH to inflammation and NASH development should next be studied in tissue-specific TGH deficient mice.

*Ldlr*^*−/−*^ mice fed WTD for 3 months develop metabolic disorders that include hyperlipidemia, obesity, insulin resistance, atherosclerosis and NASH with different etiology from *Pemt*^*−/−*^ mice. In our previous study, the loss of TGH in *Ldlr*^*−/−*^ mice ameliorated insulin resistance and atherosclerosis^37^. Because oxidized LDL plays an important role to trigger liver inflammatory response in *Ldlr*^*−/−*^ mice^25^,^52^, decreased sustained circulating LDL levels in *Tgh*^*−/−*^*/Ldlr*^*−/−*^ mice^37^ may have contributed to the ameliorated liver inflammation in this hyperlipidemic NASH model. We have previously demonstrated that *Tgh*^*−/−*^*/Ldlr*^*−/−*^ mice had lower concentration of hepatic free cholesterol than *Ldlr*^*−/−*^ mice after 3 months of WTD^37^. Free cholesterol is known to promote liver injury and NASH pathogenesis^53^,^54^,^55^. It has been reported that SREBP2 can activate NOX2 expression and enhance inflammation^56^. Decreased SREBP2 expression and pathway found in *Tgh*^*−/−*^*/Ldlr*^*−/−*^ mice^37^ may have contributed to the attenuated NASH features. In the present study, loss of TGH was also found to reduce SREBP2 pathway in 1 week HFD fed *Pemt*^*−/−*^ mice, a time period at which inflammation was first detected^22^.

In summary, roles of TGH in NAFLD progression were studied in three independent mouse models, 16-week HFD-fed obese mice, hypolipidemic and insulin sensitive PEMT deficient mice, and hyperlipidemic and insulin resistant LDLR deficient mice to represent different phases of NAFLD accompanied with different metabolic disorders. Although the pathogenesis of NAFLD in these three models is different, TGH deficiency attenuated disease progression in all the models. Therefore, pharmacological inactivation of human TGH (CES1) could potentially ameliorate fatty liver disease, and prevent progression from simple steatosis to NASH.

## Materials and Methods

### Mice

All animal experiments were approved by the University of Alberta Animal Care and Use Committee and were performed in accordance with the guidelines of the Canadian Council on Animal Care. *Tgh*^*−/−*^ mice generated previously^15^ were backcrossed on to C57BL/6J background for 10 generations. *Tgh*^*−/−*^ mice were bred with *Pemt*^*−/−*^ mice to produce *Tgh*^*−/−*^/*Pemt*^*−/−*^ mice. *Tgh*^*−/−*^/*Ldlr*^*−/−*^mice (C57BL/6J background) were generated previously^37^. All mice were maintained on a chow diet (LabDiet, PICO laboratory Rodent diet 20). At 10 weeks of age, age matched male C57BL/6J and *Tgh*^*−/−*^ mice were fed with the high-fat diet (HFD, F3282, Bio-serv, Flemington, NJ) containing 60% calories from fat for 16 weeks. Alternatively, at 10–12 weeks of age, male C57BL/6J wild-type (WT), *Tgh*^*−/−*^, *Pemt*^*−/−*^ and *Tgh*^*−/−*^/*Pemt*^*−/−*^ mice were fed with a HFD for 10 weeks. At 12 weeks of age, age matched male *Tgh*^*−/−*^/*Ldlr*^*−/−*^ and *Ldlr*^*−/−*^mice were fed with a high-fat, high-cholesterol diet (TD 88137, Harlan Teklad) containing 21% fat, 0.2% cholesterol by weight, which is usually referred to as a Western-type diet (WTD), for 12 weeks. Tail vein blood and tissues were collected from overnight fasted mice.

### Lipid and Lipidomic Analysis

Liver and plasma lipid mass was determined by either high-performance liquid chromatography (HPLC) or a kit assay (Roche Diagnostics GmbH, Mannheim, Germany), lipidomic analyses were carried out on the Acquity Ultra Performance LC and a Xevo TQ MS (Waters, Milford, MA, US) as described in the Supplementary Methods.

### Metabolic Labeling Studies

Primary hepatocytes prepared from mice were incubated overnight with serum-free DMEM. To assess hepatic *de novo* lipogenesis, hepatocytes were incubated for 4 hours in DMEM containing 10 μCi [^3^H]acetic acid and 250 μM non-radiolabeled acetic acid. Cells were harvested, lipids were extracted, resolved by thin layer chromatography (TLC), and radioactivity in various lipid classes was determined.

### RNA Isolation and Real Time qPCR Analysis

Liver RNA extraction and real time qPCR were performed using the specific primers (Supplementary Table 2) as described in the Supplementary Methods.

### Plasma Cytokine and Chemokine Assay

Mouse plasma cytokine and chemokine levels were determined using the Multiplexing LASER Bead Assay (Eve Technologies, Calgary, AB, CA).

### *In vivo* Insulin Signaling

Animals were fasted for 12 hours then 1 unit/kg body weight of human insulin or phosphate-buffered saline (PBS) was injected intraperitoneally. Fifteen minutes after injection, livers were collected. Phospho-Akt and total Akt levels in the livers were detected by immunoblotting.

### *In vivo* VLDL-TG Secretion

Mice were fasted overnight then injected intraperitoneally with Poloxamer 407 (1 g/kg body weight). Blood was collected before and after 1, 2, and 3 hours of injection. TG content was determined by a kit assay (Roche Diagnostics GmbH, Mannheim, Germany).

### Statistical Analysis

Data are presented as the mean ± SEM. Analysis was performed using the GraphPad PRISM^®^ 5 software. Statistical analysis was performed by unpaired two-tailed *t* test and one-way ANOVA followed by Bonferroni post-test. Data from studies in WT and *Tgh*^*−/−*^ mice on both chow and HFD were analyzed by two-way ANOVA followed by Bonferroni post-test. *In vivo* VLDL-TG secretion test was analyzed by two-way ANOVA followed by Bonferroni post-tests. P < 0.05 was interpreted as significantly different.

## Additional Information

**How to cite this article**: Lian, J. *et al.* Ces3/TGH deficiency attenuates steatohepatitis. *Sci. Rep.* **6**, 25747; doi: 10.1038/srep25747 (2016).

## Supplementary Material

Supplementary Information
