# Supplementary material for: Ces3/TGH Deficiency Attenuates Steatohepatitis
Source: Sci Rep. 2016 May 16;6:25747. doi: 10.1038/srep25747 (PMC4867576; doi:10.1038/srep25747)
Supplement: Supplementary Information [file srep25747-s1.pdf]

Supplementary data

Ces3/TGH Deficiency Attenuates Steatohepatitis

Jihong Lian, Enhui Wei, Jody Groenendyk, Subhash K. Das, Martin Hermansson, Lena Li, Russell Watts, Aducio Thiesen, Gavin Y. Oudit, Marek Michalak and Richard Lehner

Supplementary Methods

Lipid and lipidomic analysis

Livers were homogenized in 50 mM Tris-HCl (pH7.4), 250mM sucrose and 1mM EDTA by a glass/Teflon homogenizer for 20s, lipids were extracted by the modified Folch method [1]. Using phosphatidyldimethylethanolamine (PDME) as an internal standard, liver CE and FC were quantified by high-performance liquid chromatography (HPLC).

Lipidomic analyses were carried out on the Acquity Ultra Performance LC and a Xevo TQ MS (Waters, Milford, MA, US). For quantitation, a cocktail of internal standards for each lipid class was added to the lipid extracts. Chromatographic separations were carried out essentially as previously [2]. Identification, deisotoping and quantification of all lipid classes was performed using SECD and LIMSA [3]. Glycerophospholipid acyl residues were determined using fragmentation analyses [4,5]. SM species were annotated assuming a d18:1 long-chain base.

Liver and plasma triacylglycerol (TG) concentrations were determined by a kit (Roche Diagnostics GmbH, Mannheim, Germany) according to the manufacturers' instructions.

Hepatic free fatty acid (FFA) concentrations were determined using a kit (Wako Pure Chemical Industries, Mountain View, CA, US) according to the manufacturers' instructions.

RNA isolation and real time qPCR analysis

Livers were homogenized in Trizol Reagent (Life Technologies, CA, US) and total RNA was isolated according to the manufacturer's instruction. First-strand cDNA was synthesized from 2 µg total RNA using Superscript III reverse transcriptase (Invitrogen, CA, US) primed by oligo $(dt)_{12-18}$ and random primers (Invitrogen). Real-time qPCR was performed with the Platinum SYBR Green qPCR SuperMix-UDG kit (Invitrogen) in the StepOnePlus Real-time PCR System instrument (Life Technologies, Carlsbad, CA, US). Data were analyzed with the StepOne software. A standard curve was used to calculate mRNA level relative to that of a control gene, cyclophilin.

For the ER stress gene mRNA expression analysis, total RNA (500 ng) was used in one step RT-PCR (Bio-Rad, Hercules, CA, US) to generate cDNA for each sample. To monitor mRNA abundance, the cDNA was diluted 5-fold, with 3 µl of cDNA used in subsequent PCR reactions with primers targeting controls or selected genes. QPCR was performed using SYBR Green Master Mix (Bio-Rad) in the Rotor-Gene Q (Qiagen, Venlo, Limburg, Netherlands) instrument according to the manufacturer's instructions. Quantification was performed by expressing the threshold for each gene as a cycle number (Ct) and normalizing it to a housekeeping gene glyceraldehyde 3-phosphate dehydrogenase (GAPDH) using the equation 1/2^(Ct(gene)-Ct(gapdh)) and subsequently to the WT chow group as the control. The quantitative analysis of spliced XBP1 transcripts in mammalian cells protocol was utilized to identify mouse XBP1 specific splicing

with a pair of real-time PCR primers designed for quantification of mouse XBP1 mRNA splicing [6].

Primers of the various genes are listed in the Supplementary Table 2. All primers were synthesized at the DNA Core Facility of the University of Alberta.

Immunoblot analysis

Liver homogenates containing 30 μg protein were used for immunoblotting. For perilipin 2, proteins in liver homogenates were separated in 10% SDS polyacrylamide gel and transferred to Immun-Blot® PVDF membrane (Bio-Rad). Membranes were incubated with rabbit anti-Perilipin 2 antibody (dilution 1:1000, LSBio, Seattle, WA, US). For stearoyl-CoA desaturase-1 (SCD-1), 10% SDS polyacrylamide gel was used for protein separation. Rabbit anti-SCD1 antibody (dilution 1:1000, cell signaling, Danvers, MA, US) was used for protein detection. For the analysis of phospho-(Ser79) acetyl-CoA carboxylase (p-ACC), 8% SDS polyacrylamide gel was used for protein separation. Rabbit anti-p-ACC antibody (dilution 1:1000, cell signaling) was used for protein detection. Total ACC was blotted by peroxidase-labeled streptavidin (dilution 1:1000, KPL, Gaithersburg, MD, USA). Protein disulfide isomerase (PDI) (Dilution 1:5000, Enzo, Farmingdale, NY, US) or calnexin (dilution 1:1000, Enzo) were immunoblotted as loading controls. 10% polyacrylamide gel was utilized for immunoblotting of Ces3/TGH and PEMT. Ces3/TGH antibody was generated in our laboratory. PEMT antibody was provided by Dr. Dennis E. Vance, University of Alberta. Immunoreactivity was detected by ECL system (Amersham-Pharmacia, ON, CA) according to the manufacturer's instructions, and visualized by G:BOX system

(SYNGENE, Cambridge, UK). Immunoblots were quantified by GeneTools program (SYNGENE).

For insulin signaling analysis, proteins in liver homogenates were separated on 10% SDS polyacrylamide gel, transferred to membranes and immunoblotted for phospho-Akt (p-Akt, Ser473, dilution 1:1000) and Akt (dilution 1:1000) antibodies purchased from Cell Signaling. After densitometric analysis, the ratios between p-Akt and total-Akt were calculated in each group.

For ER stress assessment, proteins were separated in 10% SDS polyacrylamide gel. The following antibodies were used: goat anti-calreticulin (1:300, generated by Dr. Marek Michalak's laboratory), rabbit anti-tubulin (1:1000, Abcam), rabbit anti-GRP78/BiP (1:5000, Enzo, Farmingdale, NY, US), and rabbit anti-calnexin.

Histology

A portion of liver was fixed in 10% neutral buffered formalin, and 5 μm Paraffin sections were collected. For NAFLD scoring, slides were stained with hematoxylin and eosin, then steatosis, hepatocellular ballooning, portal inflammation, lobular inflammation, and fibrosis were scored by a blinded observer. The modified NAFLD activity score, which was used to assess the progression of NAFLD, is the sum of steatosis, ballooning, portal inflammation, and lobular inflammation scores [7,8].

To specifically assess liver fibrosis, Picrosirius red (PSR) staining for collagen was performed as previously described [9]. In brief, deparaffinized tissue sections were incubated with Celestain Blue for 5 min followed by washing with distilled

water and dipping in hematoxylin for 5 min. Then slides were washed with acid alcohol, dipped in Scotts tap water, incubated with 0.2% PMA (Phosphomolybdic acid) for 25 min, then dipped in PSR solution for 90 min. The stained sections were visualized by using fluorescence microscope (Olympus IX81) and data were analyzed using MetaMorph software. For the collagen volume quantification, four random fields per section, two sections per liver sample, and five livers per group were examined. Assessment was performed by a blinded observer.

Supplementary Figures

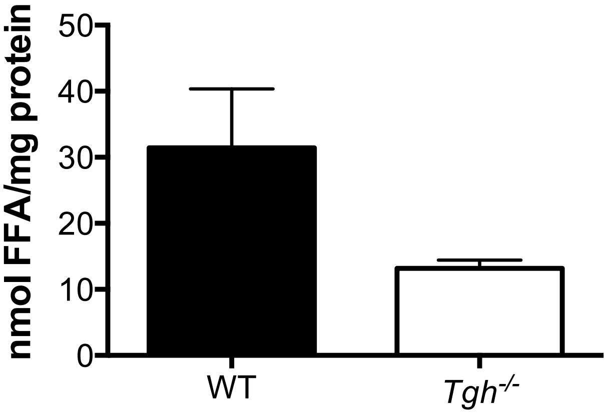

Supplementary Figure 1. Hepatic free fatty acid (FFA) concentration in WT and *Tgh*$^{-/-}$ mice after 16 weeks HFD. n=5. Data are mean ± SEM.

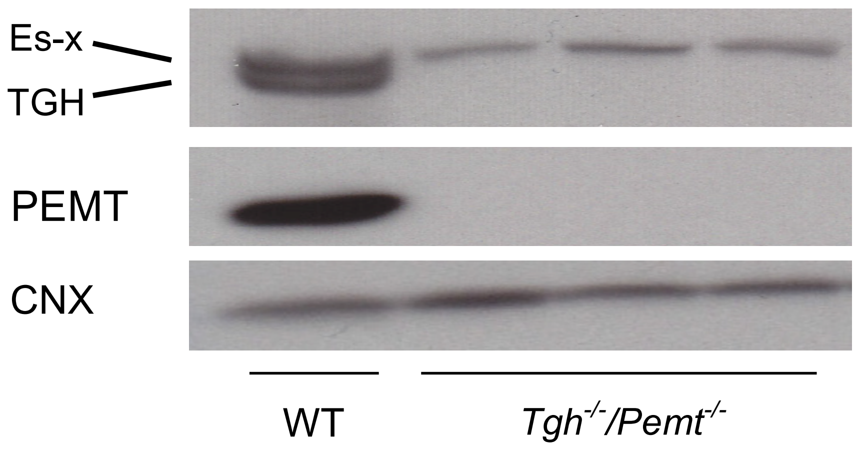

Supplementary Figure 2. Absence of TGH and PEMT in *Tgh*$^{-/-}$/*Pemt*$^{-/-}$ mice.

A

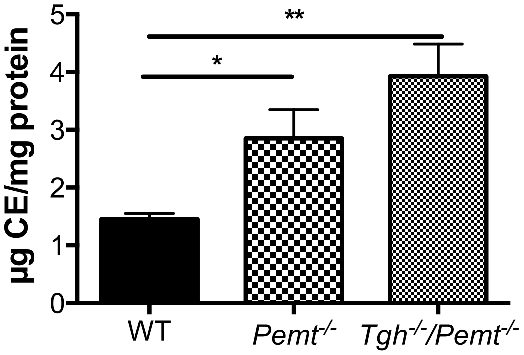

b

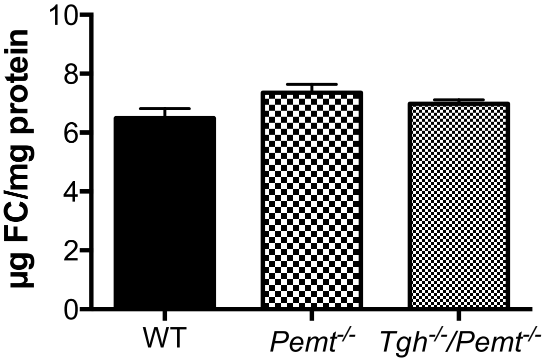

c

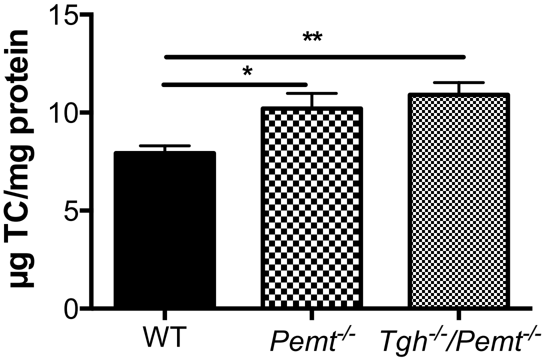

Supplementary Figure 3. **Liver cholesterol levels did not differ between *Pemt*$^{-/-}$ and *Tgh*$^{-/-}$/*Pemt*$^{-/-}$ mice after 1 week HFD.** Liver (a) cholesteryl ester

(CE), (b) free cholesterol (FC), and (c) total cholesterol (TC) mass in WT, *Pemt*$^{-/-}$, and *Tgh*$^{-/-}$/*Pemt*$^{-/-}$ mice. n=5. Data are mean ± SEM. *P<0.05, **P<0.01 vs WT control**.**

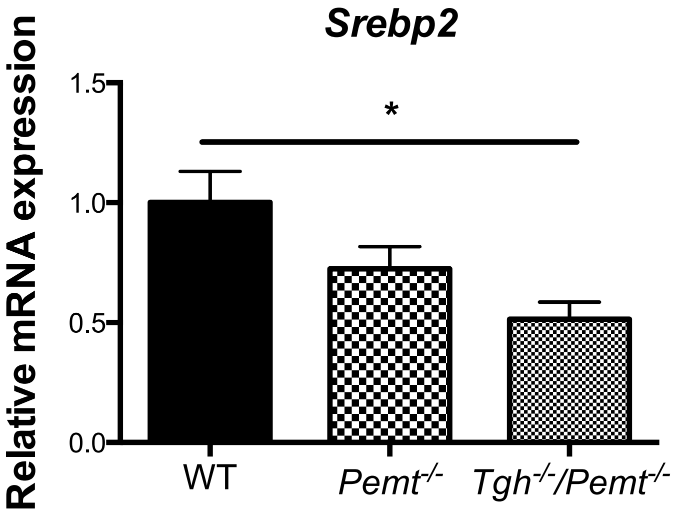

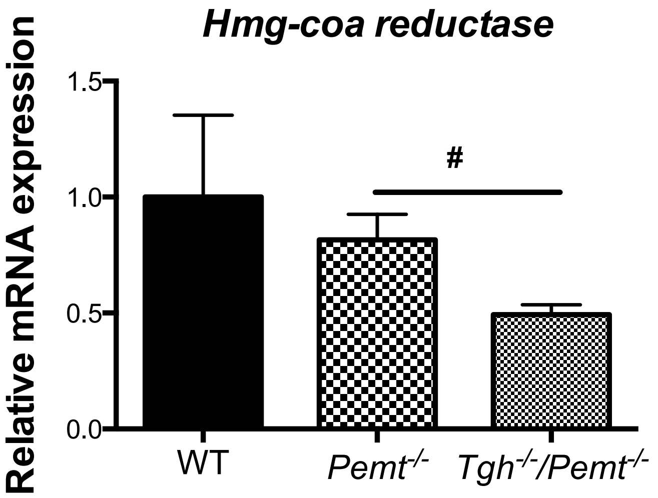

Supplementary Figure 4. qPCR analysis of SREBP2 pathway genes expression in livers of WT, *Pemt*$^{-/-}$ and *Tgh*$^{-/-}$/*Pemt*$^{-/-}$ mice after 1 week of HFD. n=5. Data are mean ± SEM. *$P<0.05$ vs WT control, $^{\#}P<0.05$ vs *Pemt*$^{-/-}$ mice.

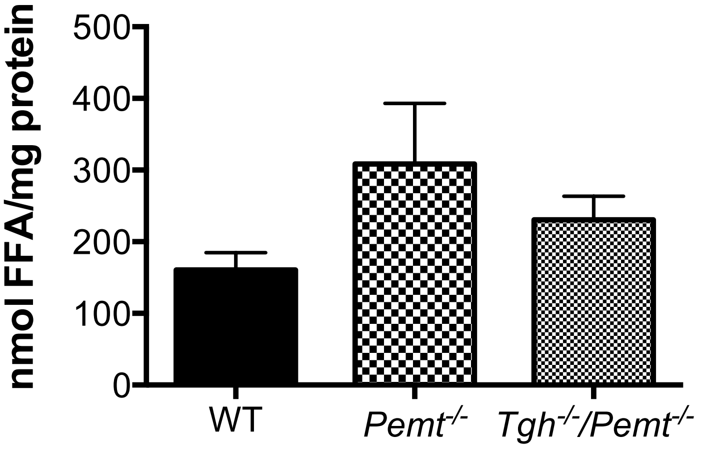

Supplementary Figure 5. Liver FFA did not differ among WT, *Pemt*$^{-/-}$, and *Tgh*$^{-/-}$/*Pemt*$^{-/-}$ mice after 16 weeks HFD. n=5-6. Data are mean ± SEM.

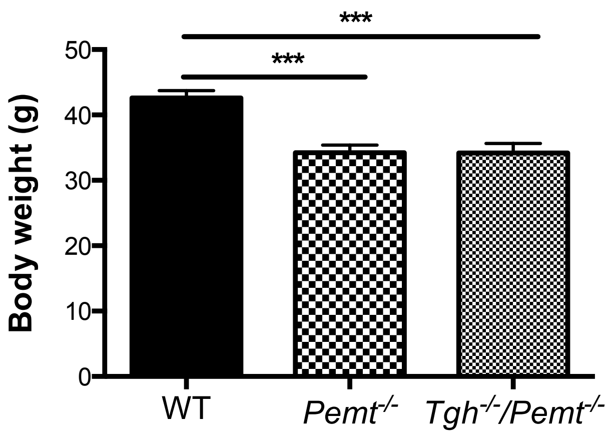

Supplementary Figure 6. Body weight of WT, *Pemt*$^{-/-}$ and *Tgh*$^{-/-}$/*Pemt*$^{-/-}$ mice after 10 weeks HFD. n=5-6. Data are mean ± SEM. ****P*<0.001 vs *Pemt*$^{-/-}$ mice.

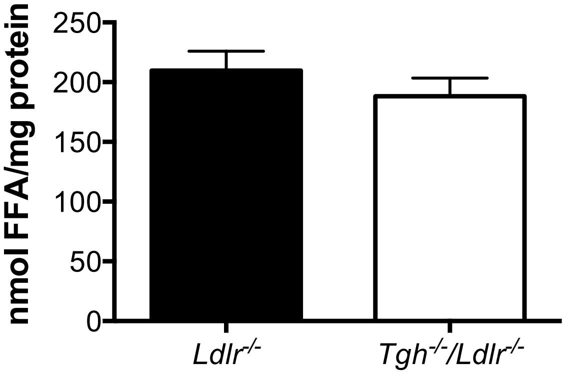

Supplementary Figure 7. Liver FFA did not differ between *Ldlr*$^{-/-}$ and *Tgh*$^{-/-}$/*Ldlr*$^{-/-}$ mice after 12 weeks WTD. n=5. Data are mean ± SEM.

a

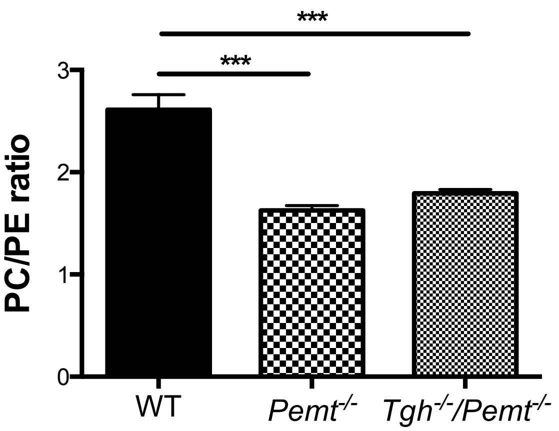

b

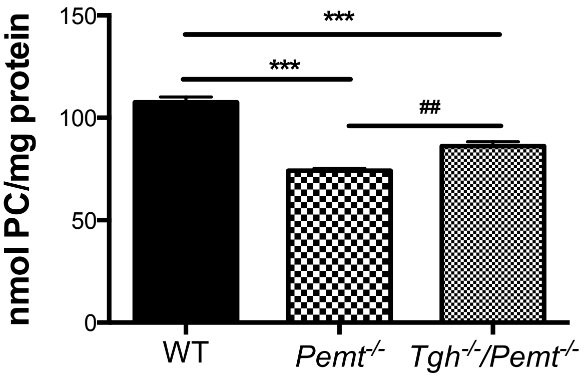

Supplementary Figure 8. Liver PC to PE ratio (a) and PC concentration (b) in WT, *Pemt$^{-/-}$* and *Tgh$^{-/-}$/Pemt$^{-/-}$* mice after 10 weeks of HFD. n=5. Data are mean ± SEM. ****P*<0.001 vs WT control, $^{\#\#}$P<0.01 vs *Pemt$^{-/-}$* mice.

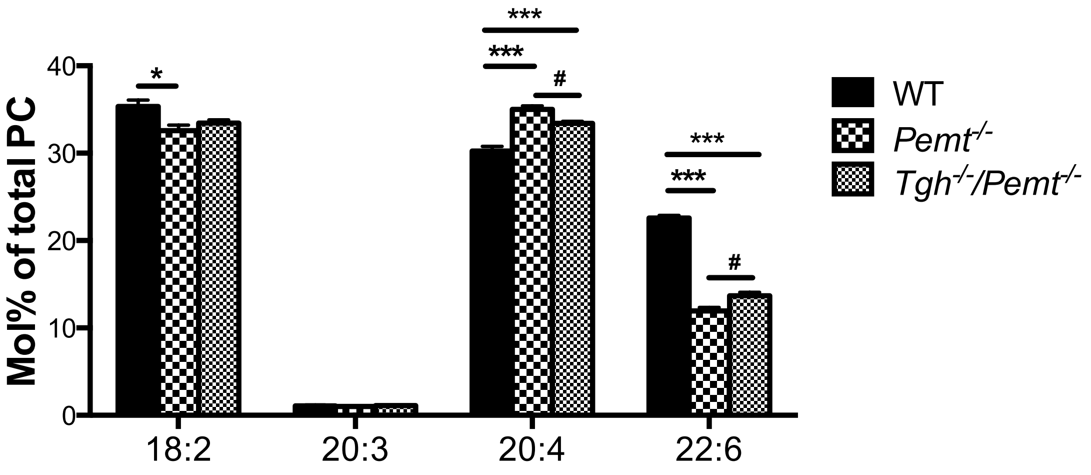

Supplementary Figure 9. PUFA compensation in WT, *Pemt$^{-/-}$*, and *Tgh$^{-/-}$/Pemt$^{-/-}$* mice after 10 weeks of HFD. n=5. Data are mean ± SEM. **P*<0.01, ****P*<0.001 vs WT control, $^{\#}$P<0.05 vs *Pemt$^{-/-}$* mice.

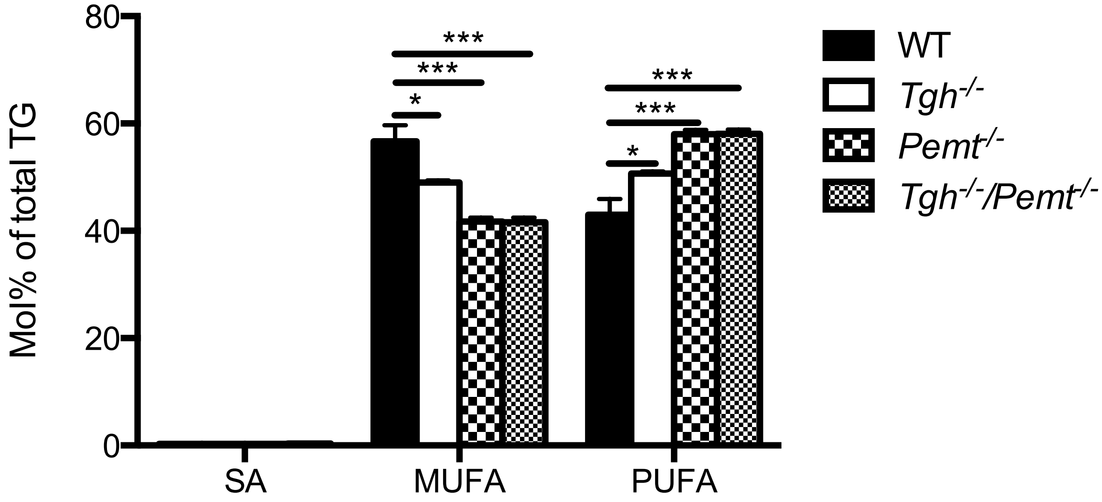

Supplementary Figure 10. Liver TG species in WT, *Tgh*$^{-/-}$, *Pemt*$^{-/-}$ and *Tgh*$^{-/-}$*/Pemt*$^{-/-}$ mice after 10 weeks of HFD. n=5. Data are mean ± SEM. **P*<0.01, ****P*<0.001 vs WT control.

a

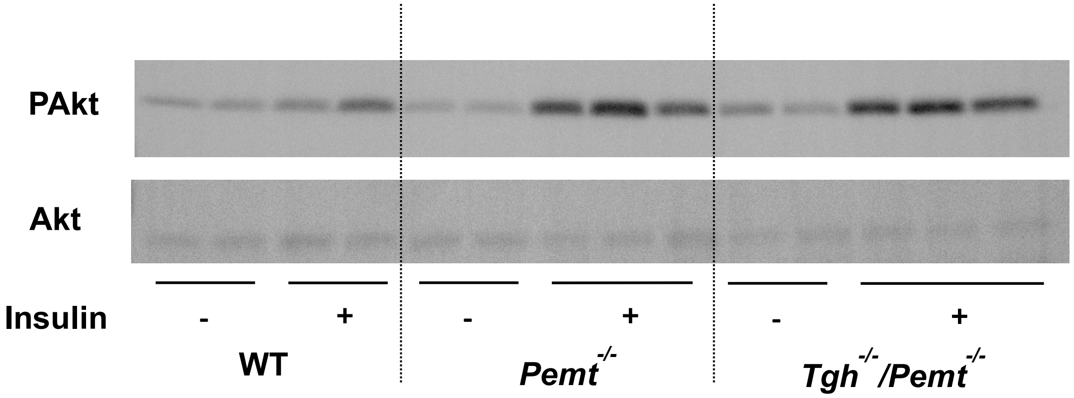

b

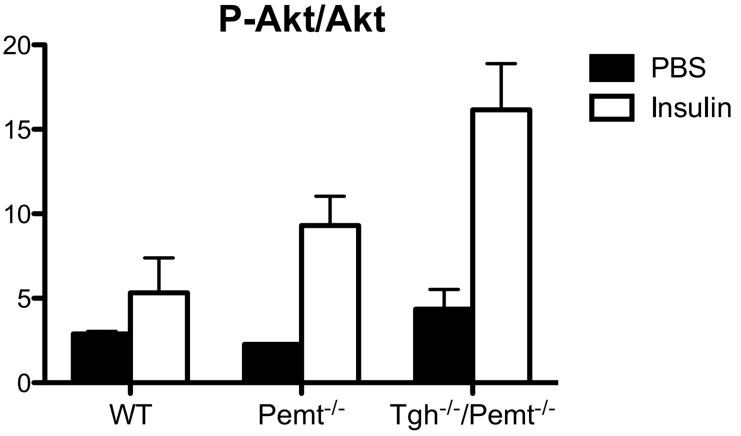

Supplementary Figure 11. ***In vivo*** **insulin signaling in WT,** ***Pemt$^{-/-}$*** **and** ***Tgh$^{-/-}$/Pemt$^{-/-}$*** **mice after 10 weeks of HFD.** Bands (a) were quantified by the densitometric analysis and the ratio of pAkt and total-Akt was calculated in each condition (b).

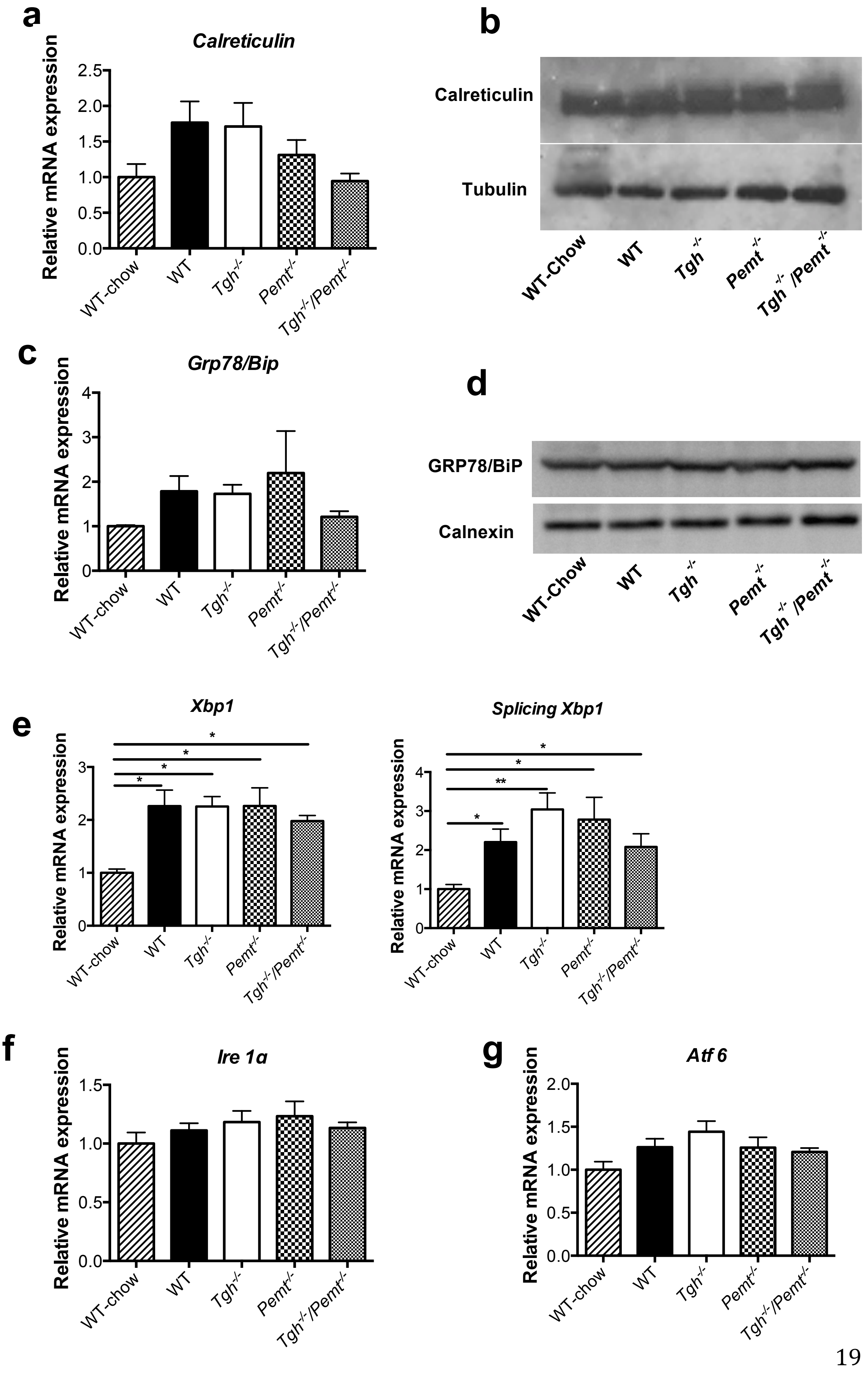
a
Calreticulin
Relative mRNA expression
2.5
2.0
1.5
1.0
0.5
0.0
WT-chow
WT
Tgh-/-
Pemt-/-
Tgh-/-/Pemt-/-
b
Calreticulin
Tubulin
WT-Chow
WT
Tgh -/-
Pemt -/-
Tgh -/- /Pemt -/-
c
Grp78/Bip
Relative mRNA expression
4
3
2
1
0
WT-chow
WT
Tgh-/-
Pemt-/-
Tgh-/-/Pemt-/-
d
GRP78/BiP
Calnexin
WT-Chow
WT
Tgh -/-
Pemt -/-
Tgh -/- /Pemt -/-
e
Xbp1
Relative mRNA expression
3
2
1
0
*
*
*
*
WT-chow
WT
Tgh-/-
Pemt-/-
Tgh-/-/Pemt-/-
Splicing Xbp1
Relative mRNA expression
4
3
2
1
0
*
*
**
*
WT-chow
WT
Tgh-/-
Pemt-/-
Tgh-/-/Pemt-/-
f
Ire 1a
Relative mRNA expression
1.5
1.0
0.5
0.0
WT-chow
WT
Tgh-/-
Pemt-/-
Tgh-/-/Pemt-/-
g
Atf 6
Relative mRNA expression
2.0
1.5
1.0
0.5
0.0
WT-chow
WT
Tgh-/-
Pemt-/-
Tgh-/-/Pemt-/-

Supplementary Figure 12. Liver ER stress markers were tested in WT, *Tgh*$^{-/-}$, *Pemt*$^{-/-}$ and *Tgh*$^{-/-}$/*Pemt*$^{-/-}$ mice after 10 weeks of HFD. WT mice fed chow diet were used as control for baseline expression of ER stress markers. (a) Expression of Calreticulin mRNA. (b) Calreticulin protein abundance determined by immunoblotting. (c) Expression of GRP78/BiP mRNA. (d) GRP78/BiP protein abundance determined by immunoblotting. (e) mRNA expression of XBP1 and spliced XBP1. (f) Expression of IRE1α mRNA. (g) Expression of ATF6 mRNA. n=5. Data are mean ± SEM. **P*<0.05, ***P*<0.01 vs WT chow control.

Supplementary Tables

Supplementary Table 1. General characteristics of the *Tgh*$^{-/-}$ mice fed chow or HFD for 16 weeks

Diet	Chow diet		HFD	
Genotype	WT	*Tgh*$^{-/-}$	WT	*Tgh*$^{-/-}$
Body weight (g)	29.4±1.55	30.5±2.10	49.5±0.54$^{\#\#\#}$	47.2±1.86$^{\#\#\#}$
Plasma TG (mg/dl)	122±14.0	68.6±6.54**	80.1±8.74$^{\#}$	61.1±10.2
Plasma CE (mg/dl)	38.1±13.1	37.4±6.99	29.0±10.6	40.4±3.48
Plasma FC (mg/dl)	109±24.5	112±19.2	69.5±26.8	98.7±28.1
Plasma NEFA (mEq/l)	1.10±0.05	0.81±0.09*	1.22±0.02	1.08±0.06$^{\#}$

Data are mean±SEM, n=5-8 in each group.

*P<0.05, **P<0.01 vs WT on the same diet condition, $^{\#}$P<0.05, $^{\#\#\#}$P<0.001 vs chow diet in the same genotype.

Supplementary Table 2. Primers used for quantitative PCR analysis

Gene	Sequence
Aco	F: 5’-CAGCAGGAGAAATGGATGCA-3’
	R: 5’-GGGCGTAGGTGCCAATTATCT-3’
Atf6	F: 5’- CAAATAGCCAACAGAAAGCCCGCA -3’
	R: 5’- TGGTTTCTGTGTACTGGACAGCCA -3’
Bip	F: 5’- GAGGATGTGGGCACGGTGGT -3’
	R: 5’- CCCTGATCGTTGGCTATGAT -3’
Calreticulin	F: 5’- AAGACTGGGATGAACGAGCCAAGA -3’
	R: 5’- AATTTGACGTGGTTTCCACTCGCC- 3’
Cd68	F: 5’- GCGGCTCCCTGTGTGTCTGAT -3’
	R: 5’- GGGCCTGTGGCTGGTCGTAG -3’
Col1a1	F: 5’- AGACATGTTCAGCTTTGTGGAC -3’
	R: 5’- GCAGCTGACTTCAGGGATG -3’
Col1a2	F: 5’- AGGACACAGTGGTATGGATGG -3’
	R: 5’- ACCTGGAGTTCCATTCTCTCC -3’
Cpt1a	F: 5’-TGAGTGGCGTCCTCTTTGG-3’
	R: 5’-CAGCGAGTAGCGCATAGTCATG-3’
Cyclophilin	F: 5’- TCCAAAGACAGCAGAAAACTTTCG -3’
	R: 5’- TCTTCTTGCTGGTCTTGCCATTCC -3’
Cyp2e1	F: 5’- GGTAATGAGGCCCGCATCCA -3’
	R: 5’- AGAGAATATCCGCAATGACA -3’
F4/80	F: 5’- CCCTCGGGCTGTGAGATTGTG -3’
	R: 5’- TGGCCAAGGCAAGACATACCAG -3’
Gapdh	F: 5’- TTCACCACCATGGAGAAGGC -3’
	R: 5’- GGCATGGACTGTGGTCATGA -3’
Hmg-coa reductase	F: 5’- TGGGCATGAACATGATCTCT -3’
	R: 5’- GGCTTCACAAACCACAGT -3’
Il-1b	F: 5’- GAAGTTGACGGACCCCAAAA -3’
	R: 5’- CCACGGGAAAGACACAGGTAG -3’
Ire1a	F: 5’- TATGCCTCTCCCTCAATGGTGCAT -3’
	R: 5’- TCAAACTTGAGGTCTGTGCTGGGA -3’
Lcad	F: 5’-GCAAAATACTGGGCATCTGAA-3’
	R: 5’-TCCGTGGAGTTGCACACATT-3’
Lox	F: 5’- TCCGACGACAACCCCTATTA -3’
	R: 5’- AGGTCCGGGAGACCGTACT -3’
Mcad	F: 5’-TTACCGAAGAGTTGGCGTATG-3’
	R: 5’-ATCTTCTGGCCGTTGATAACA-3’
Mcp1	F: 5’- CATCCACGTGTTGGCTCA -3’
	R: 5’- GATCATCTTGCTGGTGAATGAGT -3’
Nox2	F: 5’- GACTGGACGGAGGGGCTAT -3’
	R: 5’- ACTTGAGAATGGAGGCAAAGG -3’
Srebp2	F: 5’- CAGGCGACCAGGAAGAAG -3’
	R: 5’- CACGGAACTGCTGGAGAAT -3’
Xbp1	F: 5’-AAGAACACGCTTGGGAATGG-3′
	R: 5’-ACTCCCCTTGGCCTCCAC-3′
Timp1	F: 5’- GCAAAGAGCTTTCTCAAAGACC -3’

	R: 5’- AGGGATAGATAAACAGGGAAACACT -3’
Tnfa	F: 5’- GTCTACTGAACTTCGGGGTGA -3’
	R: 5’- CACCACTTGGTGGTTTGCTACGAC -3’
Tgf1b	F: 5’- CGCCATCTATGAGAAAACCA -3’
	R: 5’- CCAAGGTAACGCCAGGAAT -3’
Ucp2	F: 5’-TTACCGAAGAGTTGGCGTATG-3’
	R: 5’-ATCTTCTGGCCGTTGATAACA-3’
Spliced Xbp1	F: 5′- GAGTCCGCAGCAGGTG -3′
	R: 5′- GTGTCAGAGTCCATGGGA -3′

Supplemental Reference

1. Folch, J., Lees, M. & Sloane Stanley, G.H. A simple method for the isolation and purification of total lipides from animal tissues. *J Biol Chem* **226**, 497-509 (1957).
2. Zhao, Y.Y., Xiong, Y. & Curtis, J.M. Measurement of phospholipids by hydrophilic interaction liquid chromatography coupled to tandem mass spectrometry: the determination of choline containing compounds in foods. *J Chromatogr A* **1218**, 5470-5479 (2011).
3. Haimi, P., Uphoff, A., Hermansson, M. & Somerharju, P. Software tools for analysis of mass spectrometric lipidome data. *Anal Chem* **78**, 8324-8331 (2006).
4. Kainu, V., Hermansson, M. & Somerharju, P. Electrospray ionization mass spectrometry and exogenous heavy isotope-labeled lipid species provide detailed information on aminophospholipid acyl chain remodeling. *J Biol Chem* **283**, 3676-3687 (2008).
5. Ekroos, K.*, et al.* Charting molecular composition of phosphatidylcholines by fatty acid scanning and ion trap MS3 fragmentation. *J Lipid Res* **44**, 2181-2192 (2003).
6. Back, S.H., Schroder, M., Lee, K., Zhang, K. & Kaufman, R.J. ER stress signaling by regulated splicing: IRE1/HAC1/XBP1. *Methods* **35**, 395-416 (2005).
7. Brunt, E.M., Janney, C.G., Di Bisceglie, A.M., Neuschwander-Tetri, B.A. & Bacon, B.R. Nonalcoholic steatohepatitis: a proposal for grading and staging the histological lesions. *Am J Gastroenterol* **94**, 2467-2474 (1999).
8. Kleiner, D.E.*, et al.* Design and validation of a histological scoring system for nonalcoholic fatty liver disease. *Hepatology* **41**, 1313-1321 (2005).
9. Wang, Z.*, et al.* TIMP2 and TIMP3 have divergent roles in early renal tubulointerstitial injury. *Kidney Int* **85**, 82-93 (2014).
